# Endotoxin Tolerance Impinges on T Cell Activation and Chemoattraction in Autoimmune Diabetes

**DOI:** 10.1155/jimr/7395567

**Published:** 2026-04-24

**Authors:** Satu M. Silojärvi, Linda A. A. Leino, Sakari A. Pöysti, Arno L. M. Hänninen

**Affiliations:** ^1^ Institute of Biomedicine, University of Turku, Turku, Finland, utu.fi; ^2^ TYKS Laboratories, Clinical Microbiology, Turku University Hospital, Turku, Finland, vsshp.fi

**Keywords:** autoimmune diabetes, endotoxin tolerance

## Abstract

Gut microbiota may affect the development of autoimmune (type 1) diabetes by differential potency of intestinal species to induce endotoxin tolerance (ET). However, where ET impinges on immune mechanisms underlying autoimmune diabetes is yet incompletely understood. We investigated the effects of lipopolysaccharide (LPS) from *E. coli* and *B. vulgatus*, two common intestinal species dominating either in low‐ or high‐incidence countries, on activation and chemoattraction of islet‐specific T cells in non‐obese diabetic (NOD) mice. Intraperitoneal (i.p.) injection of *E. coli* LPS induced costimulatory ligands CD40, CD80 and CD86 on both conventional and cross‐presenting (XCR1+) dendritic cells (DC) and islet‐specific glucose‐6‐phosphatase catalytic subunit‐related protein (IGRP)‐reactive islet‐specific T cells in the pancreatic lymph node. In comparison to mice not primed with *E. coli* LPS or primed with *B. vulgatus* LPS, the second injection of *E. coli* LPS lowered the frequency of IGRP‐reactive T cells and CD80 expression on DC subsets, as well as CD44 and CD69 activation markers and the CXCR3 chemokine receptor on IGRP‐reactive T cells. In islets, expression of chemokine CXCL10 accentuated, and insulitis became more severe in mice primed with *B. vulgatus* LPS. Our results provide mechanistic insight into how ET affects islet autoimmunity and suggest that physiological exposure to *E. coli* LPS may benefit in moderating autoimmune diabetes.

## 1. Introduction

Type 1 diabetes (T1D) is an autoimmune disease resulting in T cell‐mediated destruction of pancreatic islet β‐cells. Although genetic factors predispose to T1D, disease onset and progression are likely triggered by environmental factors. Improved sanitation and lower incidence of early childhood infections are seen as potential triggers or drivers of autoimmune diseases such as T1D [1, 2]. Proposedly, the hygiene hypothesis explains this by linking certain microorganisms with immune system education and regulation [3]. Limited exposure to bacteria and low diversity in gut microbiota may predispose to immune reactions against self‐antigens, including islet autoantigens in T1D [4]. Studies in genetically susceptible mice demonstrated that perturbations in early‐life intestinal microbiota accelerate T1D, while restoration of microbiota counteracts this acceleration [5]. Similarly, antibiotic‐induced dysbiosis predisposes to allergies in mice [6]. However, the microbes and immunological mechanisms behind these phenomena are still incompletely understood.

One mechanism through which different species of gut microbiota can affect immune education is stimulation and tolerization through microbial components such as lipopolysaccharides (LPS). Microbial components signal through pattern recognition receptors (PRRs) such as Toll‐like receptors (TLRs), and LPSs have been studied as mediators of immune tolerance [7, 8] and autoimmune diseases [9, 10]. Endotoxin tolerance (ET), an adaptive reprogramming of immune cells, refers to a state in which cells or organisms diminish their immune response to further challenges once exposed to LPS [11], altering inflammatory mechanisms. Molecular mechanisms of ET include downregulation of the endotoxin receptor TLR4 [12], and downstream of TLR4, regulation of MyD88‐ and TRIF‐dependent pathways through negative regulators IRAK‐M and A20, also influencing the development of ET [11, 13]. ET is mostly measured and characterised as diminished production and secretion of tumour necrosis factor alpha (TNFα) [14], although in addition, many interleukins and chemokines are also downregulated [15]. Downregulation of costimulatory ligands has been documented in human monocytes [15, 16], reducing proliferation and interferon gamma (IFNγ) production of T cells [16], suggesting that ET influences adaptive immune responses. While ET protects against hyperinflammation and autoimmune diseases, increasing evidence suggests that it may also impair immunosurveillance and promote infections as well as some diseases such as cancer [17].

The adverse effects of gut microbiota dysbiosis in T1D are supported by both clinical studies and studies in animal models. Clinical studies have reported an association between islet autoimmunity and increased abundance of the phylum *Bacteroidetes* or genus *Bacteroides* (reviewed in [18, 19]), whereas in animal models, even single bacterial species may confer favourable immune system alterations and protection from diabetes [20, 21]. A seminal study [22] demonstrated that LPS derived from *E. coli* is superior to LPS from *B. vulgatus* in inducing ET, owing to structural diversity likely related to the number of acyl chains in the lipid A component of LPS [23, 24]. While in vitro ET was determined using human mononuclear cells, it was also reflected in delayed development of hyperglycaemia in prediabetic non‐obese diabetic (NOD) mice. Although ET determined in vitro may reflect tolerization of myeloid cells in vivo, its role in antigen presenting and costimulatory activity of dendritic cells (DCs) involved in activation of islet‐specific T cells has not been addressed in vivo.

To elucidate how LPSs from *E. coli* and *B. vulgatus* modify facets of autoimmunity driving β‐cell destruction, we determined costimulatory markers in conventional and cross‐presenting DCs and activation of islet‐specific T cells in pancreatic lymph nodes (Figure S1). We used LPS itself as the challenge to determine ET, since it covers all aspects of ET downstream of TLR4.

## 2. Results

### 2.1. *E. coli* LPS Moderates Peritoneal Cell TNFα Production

To evaluate the immunostimulatory potential of structurally different LPSs, peritoneal cells of naïve NOD mice were stimulated with LPS prepared from *B. vulgatus* or *E. coli*. Stimulation was performed in vitro with a single exposure to LPS, and TNFα production was analysed from the supernatant (Figure 1A). Both LPS preparations induced TNFα production in peritoneal cells, while *B. vulgatus* LPS elicited a significantly weaker response compared to *E. coli* LPS. In unstimulated cells, TNFα production was negligible.

Figure 1TNFα production by murine peritoneal cells. *E. coli* LPS is more efficient in inducing TNFα production by peritoneal cells than *B. vulgatus* LPS (A). After pretreatment with *E. coli* LPS, peritoneal cells were rendered tolerant to challenge with zymosan (B) and commercial *E. coli* LPS (C). When challenged with commercial *E. coli* LPS, also pretreatment with *B. vulgatus* LPS showed a trend towards diminished TNFα production in response (C). Combined data from five independent experiments (*n* = 7–9), unpaired two‐tailed Student’s *t*‐test (normally distributed data) or Mann–Whitney *U* test (A). Combined data from three independent experiments (*n* = 5–6), one‐way ANOVA with Tukey’s multiple comparison (normally distributed data) or Kruskal–Wallis test with Dunn’s multiple comparison (B,C). Bars indicate mean ± SEM,  ^∗^ *p*  < 0.05;  ^∗∗^ *p*  < 0.01;  ^∗∗∗^ *p*  < 0.001;  ^∗∗∗∗^ *p*  < 0.0001.

**Figure:**
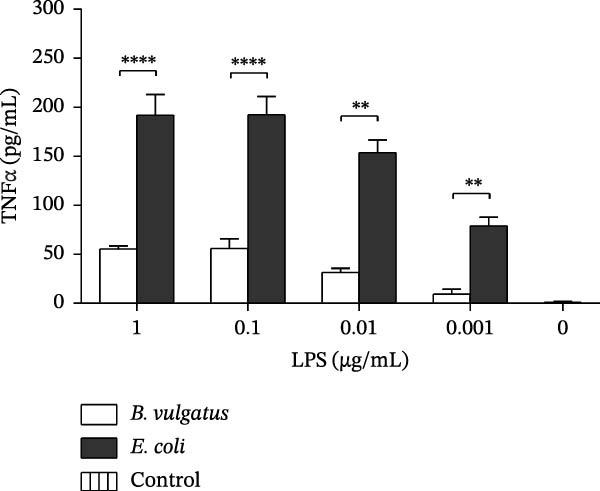
(A)

**Figure:**
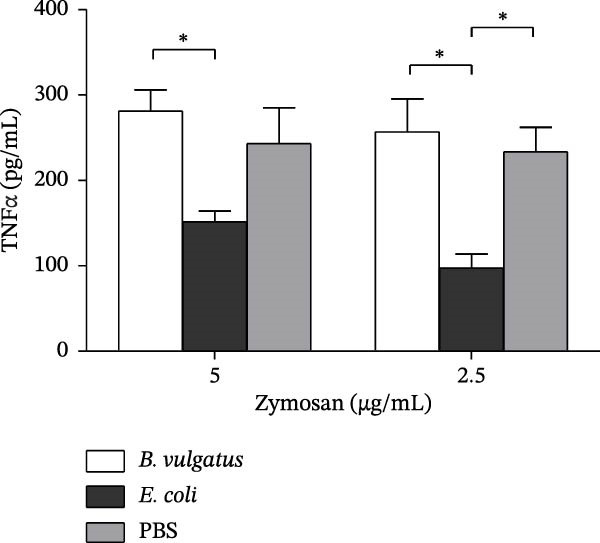
(B)

**Figure:**
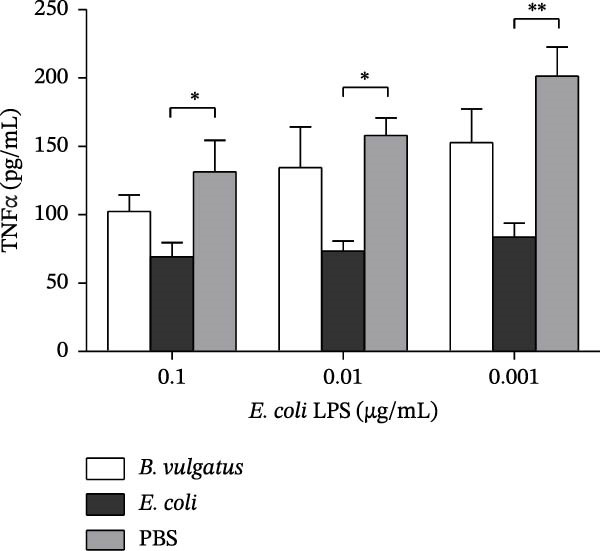
(C)

As the LPSs prepared from *B. vulgatus* and *E. coli* differed in their potency to induce TNFα in peritoneal cells, we studied whether they differed in their capability to induce ET, that is, tolerance to a subsequent challenge with LPS. Tolerance was modelled by intraperitoneal (i.p.) injections of *B. vulgatus* or *E. coli* LPS for priming and by subsequently challenging peritoneal cells in vitro with zymosan or commercial *E. coli* LPS and measuring TNFα production thereafter. Priming of mice in vivo with *E. coli* LPS, but not *B. vulgatus* LPS, rendered peritoneal cells tolerant to in vitro challenge by zymosan and commercial *E. coli* LPS. (Figure 1B,C, respectively).

### 2.2. *E. coli* LPS Moderates CD80 and CD86 Expression in PaLN DCs

We first investigated whether *E. coli* and *B. vulgatus* LPS differed in their capability to activate DCs. PaLN DCs were studied after i.p. injection with *E. coli* or *B. vulgatus* LPS, and cell populations were evaluated after 24 h by flow cytometry. Both conventional (CD11c+ MHCII+) and cross‐presenting (CD11c+ MHCII+ XCR1+) DCs were studied separately for their expression of costimulatory markers CD40, CD80 and CD86. Compared to *B. vulgatus* LPS and control, exposure to *E. coli* LPS resulted in upregulation of all studied markers, represented by the higher mean fluorescent intensity (MFI) values (Figure 2A). In contrast, *B. vulgatus* LPS did not differ from control.

Figure 2Expression of activation markers on conventional and cross‐presenting PaLN DCs following exposure to *B. vulgatus* LPS or *E. coli* LPS. Exposure to *E. coli* LPS results in significant upregulation of activation and cross‐presentation markers at 24 h (A), whereas second exposure to *E. coli* LPS shows reduced marker intensity in PaLN dendritic cells (B). Combined data from two independent experiments (*n* = 5), one‐way ANOVA with Tukey’s multiple comparison (normally distributed data) or Kruskal–Wallis test with Dunn’s multiple comparison. Bars indicate mean ± SEM,  ^∗^ *p*  < 005;  ^∗∗^ *p*  < 0.01;  ^∗∗∗^ *p*  < 0.001.

**Figure:**
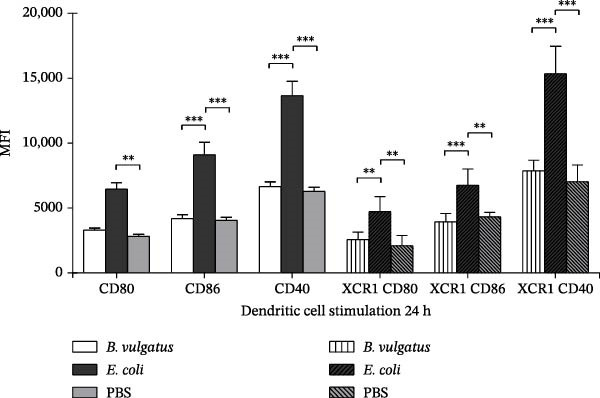
(A)

**Figure:**
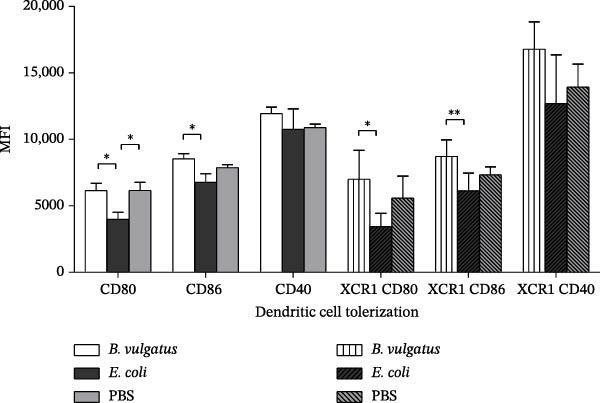
(B)

Because LPS subtypes were not equivalent in tolerizing peritoneal cells, we studied whether this was true also for DCs in PaLN. For priming and for challenge, NOD mice were first injected with *E. coli* or *B. vulgatus* LPS and subsequently challenged with *E. coli* LPS as above. Activation status was studied in both conventional and cross‐presenting PaLN DCs based on expression of CD80, CD86 and CD40. In both DC‐subtypes, the levels of CD80 and CD86 were significantly lower in *E. coli* LPS‐primed group compared to the *B. vulgatus*‐primed group (Figure 2B). Thus, DCs were tolerized when primed with *E. coli* LPS, whereas *B. vulgatus* LPS did not induce tolerance to subsequent LPS challenge.

### 2.3. *E. coli* LPS Moderates Activation of Islet‐Specific Glucose‐6‐Phosphatase Catalytic Subunit‐Related Protein (IGRP)‐Specific T Cells

We investigated whether differences in activation of islet‐specific T cells were apparent following induction of ET. Again, tolerance was induced with *E. coli* or *B. vulgatus* LPS. To this end, we determined the quantities of IGRP‐tetramer reactive T cells and their expression of activation markers CD69 and CD44. In addition, the migratory potential of diabetogenic T cells was evaluated by expression levels of the C‐X‐C motif chemokine receptor 3 (CXCR3). The percentage of IGRP‐tetramer‐positive cells among CD8+ T cells was significantly lower in mice exposed to *E. coli* LPS (Figure 3B), while exposure to *B. vulgatus* LPS did not lower their percentage when compared to control. Their CD44 expression was significantly higher in mice exposed to *B. vulgatus* LPS compared to control (Figure 3C) and CD69 expression was also higher, although not significantly (Figure 3D). Furthermore, the percentage of CXCR3 positivity in IGRP‐tetramer‐positive T cells was lower in mice exposed to *E. coli* LPS as opposed to mice exposed to *B. vulgatus* LPS (Figure 3E). In CD69 data, one staining failed, resulting in the exclusion of 1 + 1 + 3 mice, and in CXCR3 data, 0 + 1 + 1 mice were detected as outliers with Tukey’s 1.5 IQR method and excluded from results.

Figure 3Activation of T cells in pancreatic lymph node. T cell populations were analysed after two doses of *B. vulgatus* or *E. coli* LPS or PBS (A). The percentage of IGRP+/CD8+ T cells was significantly lower in mice exposed to *E. coli* LPS compared to *B. vulgatus* LPS (B), the mean control tetramer level is indicated with a dashed line. Significantly elevated expression of CD44 (C) and slightly elevated expression of CD69 (D) in IGRP+ T cells of mice exposed to *B. vulgatus* LPS was detected. The percentage of CXCR3 in IGRP+ T cells was significantly lower following *E. coli* LPS injections compared to *B. vulgatus* LPS (E). Combined data from three independent experiments, symbols represent one individual mouse and lines indicate the mean. One‐way ANOVA with Tukey’s multiple comparison (normally distributed data) or Kruskal–Wallis test with Dunn’s multiple comparison,  ^∗^ *p*  < 0.05;  ^∗∗^ *p*  < 0.01.

**Figure:**
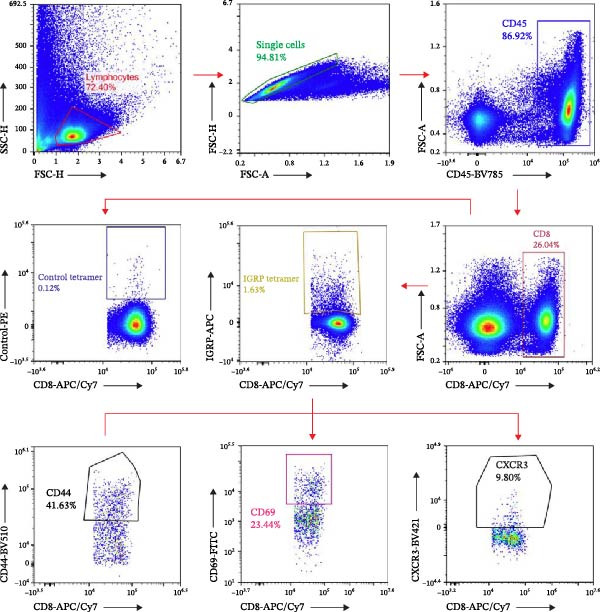
(A)

**Figure:**
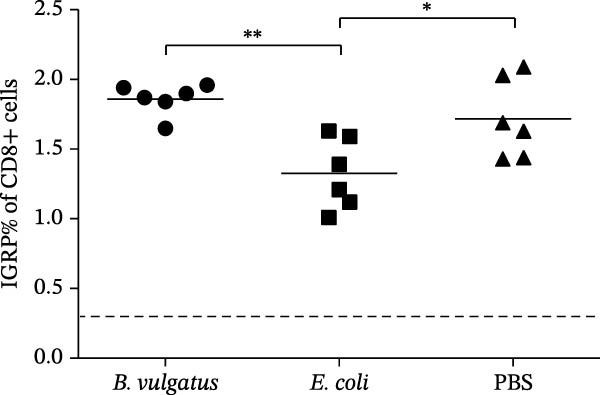
(B)

**Figure:**
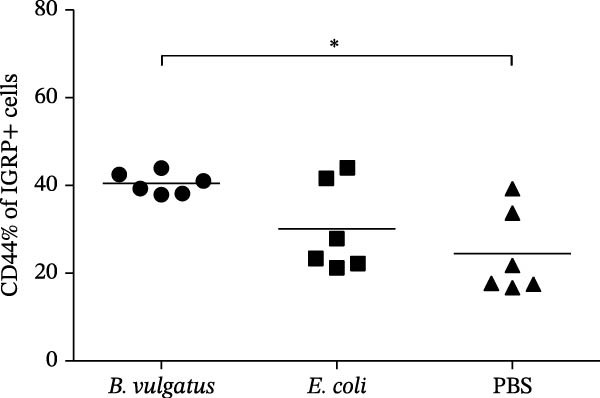
(C)

**Figure:**
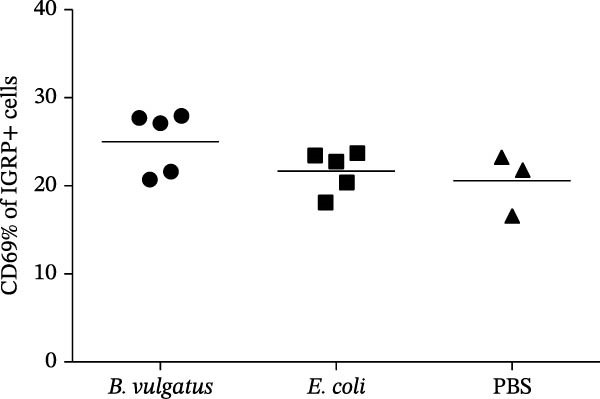
(D)

**Figure:**
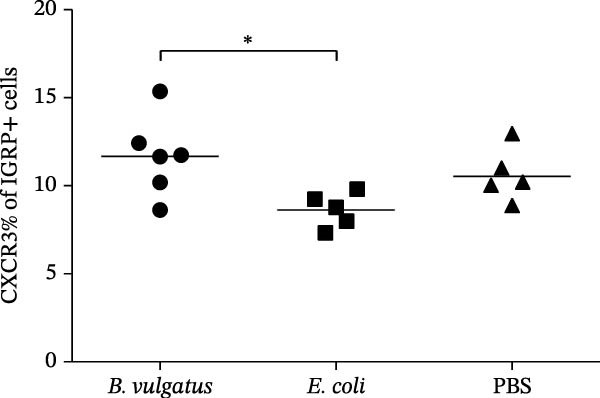
(E)

### 2.4. *B. vulgatus* LPS is Inferior to *E. coli* LPS in Stimulating and Tolerizing CXCL10 Expression in Islets

**Figure 4:**
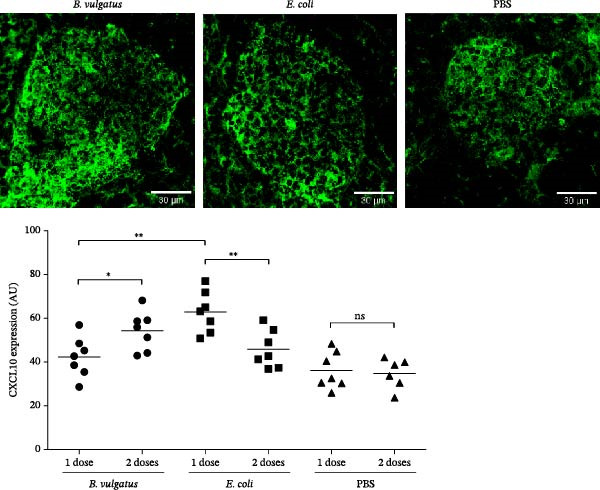
Pancreatic islet CXCL10 expression. CXCL10 levels in islets were determined after one (stimulation) or two (tolerance) doses of LPS. CXCL10 expression in intact pancreatic islets (green = CXCL10) after induction with two doses of *E. coli* or *B. vulgatus* LPS (A) or PBS. CXCL10 levels increase significantly after two doses of *B. vulgatus* LPS, while with *E. coli* LPS CXCL10 expression decreases after second dose (B). *E. coli* LPS was more efficient at stimulating CXCL10 expression compared to *B. vulgatus* LPS. Combined data from 2 to 3 independent experiments, symbols represent one individual mouse and lines indicate the mean. Unpaired two‐tailed Student’s *t*‐test,  ^∗^ *p*  < 0.05;  ^∗∗^ *p*  < 0.01.

To determine whether *E. coli* and *B. vulgatus* LPS differ in their capability to induce C‐X‐C motif chemokine ligand 10 (CXCL10) expression in pancreatic islets, we injected intraperitoneally *E. coli* or *B. vulgatus* LPS to NOD mice and studied CXCL10 expression 72 h later. When compared to *B. vulgatus* LPS, mice injected with *E. coli* LPS expressed significantly higher levels of CXCL10 in healthy islets (Figure 4).

As *E. coli* LPS induced tolerance in cells of the innate and adaptive immune systems more efficiently than *B. vulgatus* LPS, we investigated if this difference is also evident regarding islet CXCL10 expression. Tolerance was again modelled by dosing LPS two times. After two doses, CXCL10 levels were significantly higher in *B. vulgatus* LPS group and significantly lower in *E. coli* LPS group when compared to the first LPS dose in the respective groups (Figure 4).

### 2.5. *B. vulgatus* LPS Accelerates Islet Destruction in NOD Mice

Finally, to test the impact of LPS‐driven immune tolerance in islet autoimmunity, we assessed the amount of infiltrating cells in the pancreatic islets (insulitis). Repeated injection of *B. vulgatus* LPS resulted in notably higher levels of insulitis (Figure 5A,B) when compared to injection of *E. coli* LPS.

Figure 5Insulitis levels in pancreatic islets. The amount of infiltrating cells is increased by *B. vulgatus* LPS (A), and differences are detected in mean insulitis scores between groups (B). Combined data from two independent experiments, symbols represent one individual mouse and lines indicate the mean. One‐way ANOVA with Tukey’s multiple comparison.

**Figure:**
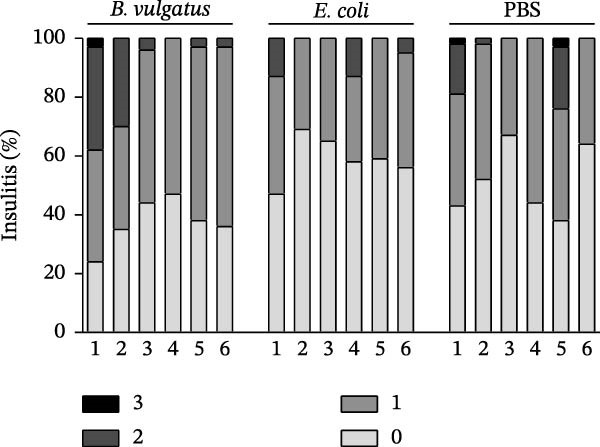
(A)

**Figure:**
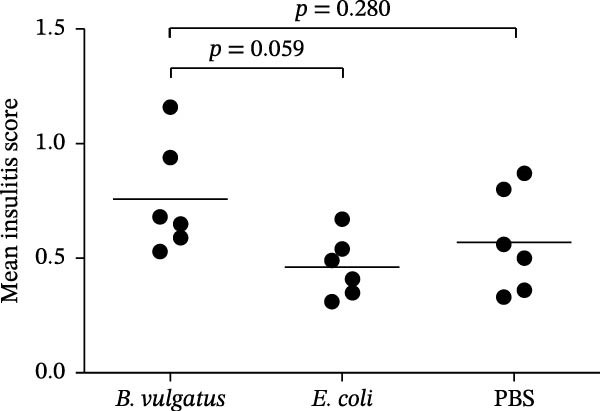
(B)

## 3. Discussion

Growing evidence indicates a connection between gut microbiota and T1D‐related autoimmunity [4, 25, 26], but the pathophysiological mechanisms mediating this effect are incompletely understood. One mechanism linking microbiota composition to T1D risk proposedly derives from differences in the molecular composition of LPSs between Gram‐negative bacteria [22]. Molecular characterisation of LPS in *B. vulgatus* and *E. coli* identified differences in the lipid A part of LPS owing to variation in acyl chains in the lipid A moiety of the LPS molecule [22–24], and their purified LPS molecules showed remarkable differences in their ability to induce ET in monocytes [22]. In this study, we focused on the relevance of ET in the presentation of islet antigens to T cells and their imprinting of chemokine receptors endowing their activation and homing to pancreatic islets. Our results show that in vivo‐induced ET affects antigen‐presenting cells and T cells in pancreatic lymph nodes, and the expression of T1D‐associated chemoattractant CXCL10 in endocrine cells of pancreatic islets [27, 28].

Earlier studies indicate that both murine splenocytes and primary human monocytes are tolerized against TNFα secretion by conditioning the cells with *E. coli* LPS [22]. In these studies, TNFα secretion was determined following a zymosan challenge, and zymosan acts foremost through TLR2 [29]. To investigate if ET also affects the signalling pathway downstream of TLR4, we determined if TNFα release following *E. coli* LPS challenge is attenuated similarly. Priming with *E. coli* LPS attenuated TNFα release to an equal level irrespective of the differences in their mechanism of action.

DCs are essential in initiating the autoimmune pathology against β‐cells, as they present β‐cell antigens to autoreactive T cells in PaLN [30], and depletion of these cells protects from the development of T1D [31, 32]. It is still under debate how gut and gut‐derived signals affect islet autoimmunity, but DCs are known to sample antigens in the gut [33]. In addition, evidence suggests that a lymphatic route may exist between the gut and PaLN [34]. We therefore investigated the different capacities of *B. vulgatus* and *E. coli* LPS to activate DCs in PaLN. In line with studies on human monocyte‐derived DCs [22] and murine bone marrow‐derived DCs (BMDCs) [35], *E. coli* LPS proved to stimulate DCs in PaLN more efficiently, as expression of studied surface markers was significantly upregulated in *E. coli* LPS‐treated mice compared to *B. vulgatus* LPS. *E. coli* LPS was also superior in tolerizing both DC subsets, characterised by reduced expression of DC activation markers in the *E. coli* LPS group.

To further validate the relevance of ET in T cell‐mediated islet autoimmunity [36–38], we studied whether the differences in LPS derived from *E. coli* and *B. vulgatus* pose different effects on T lymphocyte activation as well. We therefore studied islet‐specific CD8+ T cells specific for IGRP [39] and found that mice repeatedly exposed to *E. coli* LPS had a significantly lower percentage of IGRP‐specific T cells among CD8+ T cells compared to *B. vulgatus* LPS and the control. These results suggest that following ET, the diminished DC activation also extends to the expansion of islet‐specific CD8+ T cells in vivo, which has a crucial role in T1D pathogenesis [40, 41], as the ablation of IGRP+/CD8+ T cells confers strong disease protection in NOD mice [42–44]. Most likely, the fate of the naïve IGRP+ T cells is dictated by a lower level of costimulation, leading to apoptosis (death by neglect) or anergy after encounter of DC downregulating CD80/CD86 following LPS tolerance induction.

Since CXCR3+ T cells have been detected in PaLN of NOD mice [45] and in islets of T1D patients [46], we studied the expression of CXCR3 on IGRP+/CD8+ T cells. CXCR3 was expressed less in mice tolerized with *E. coli* LPS, suggesting they also differ in their potential to migrate into islets along the CXCL10 gradient. To our knowledge, no previous studies exist focusing on the effects of various LPS subtypes on the induction of tolerance in islet‐specific T lymphocytes, or T cells in general. Also, infection with *C. rodentium* increases the activation and proliferation of islet‐specific CD8+ and CD4+ T cells in the PaLN of mice [34], suggesting that pancreatic lymph nodes are prone to gut‐derived microbial and other inflammatory signals.

Pancreatic β‐cells have been shown to produce CXCL10 under proinflammatory conditions [47], and increased islet expression of CXCL10 has been reported both in T1D patients and animal models [48, 49]. Moreover, we have shown that CXCL10 is induced in islet‐resident myeloid cells by LPS independently of IFNγ, which suggests that islets recognise the elevated plasma LPS levels through TLR4 and subsequently release CXCL10 [28], potentially leading to the attraction of diabetogenic T cells expressing CXCR3.

Perhaps most importantly, islet‐specific CD4+ and CD8+ T cells are attracted into healthy islets by the chemokine CXCL10 [27, 28, 47]. Our results are in line with a previous study of stimulation of human peripheral blood monocyte‐derived DCs and murine bone marrow monocyte‐derived macrophages [50]. Corresponding to mechanisms in the formation of tolerance [13], CXCL10 levels in pancreatic islets free of insulitis were even higher after two doses of *B. vulgatus* LPS, whereas the second dose of *E. coli* LPS downregulated CXCL10 expression. Through decreased CXCL10 expression, *E. coli* LPS may decrease T cell migration into islets and thus destruction of islet β‐cells. Results regarding the insulitis are comparable with the previous study [22], suggesting that *B. vulgatus* LPS does neither increase the islet destruction, nor reduce it as *E. coli* LPS.

Gut microbial communities affect epithelial barrier function, as is shown by the gut symbiont *Akkermansia muciniphila* [51], which also delays diabetes onset in NOD mice. In addition to intestinal barrier integrity [20, 28], ET plays a role in attenuating islet‐specific autoimmunity, but the source of LPS matters. Our findings support the notion that together with a compromised gut barrier function, leakage of LPS from the gut may promote islet autoimmunity by attenuating the establishment of ET.

## 4. Materials and Methods

### 4.1. Bacterial Culture

*B. vulgatus* and *E. coli* strains isolated from human faecal sample, annotated by 16S‐rDNA‐sequencing, were thawed, and both liquid and solid cultures were established in fastidious anaerobe broth (FAB) and blood agar plates, as well as in Luria–Bertani (LB) broth and LB agar plates, respectively. *B. vulgatus* was cultured in anaerobic conditions (0% O_2_ 5% CO_2_, +37°C) for 48 h and *E. coli* for 16 h, +37°C.

### 4.2. LPS Extraction

LPS was extracted with the LPS Extraction Kit (Abcam, ab239718) according to the manufacturer’s protocol. Extracted LPS was quantified with phenol–sulphuric acid detection method. Commercial LPS from *E. coli* 055:B5 (Difco Laboratories) was used as a standard.

### 4.3. Mice

NOD (ShiLtJ) mice were purchased from Jackson Laboratory and maintained in the University of Turku Central Animal Laboratory (UTUCAL). The animals were housed in individually ventilated cages (IVC) under controlled conditions with a temperature 21 ± 3°C, relative humidity 55 ± 15% and 12/12 h light cycle. The mice were fed *ad libitum* with the CRM(E) diet (Special Diet Services, 324 Witham, Essex, England), and water was provided *ad libitum*. Animal experimentation was approved by the National Animal Experiment Board of Finland (License Number: ESAVI/19866/2019) in accordance with the EU (Directive 2010/63/EU).

### 4.4. TNFα Production by Peritoneal Cells

Peritoneal cells were obtained from naïve NOD mice of both sexes by peritoneal lavage. In total 5 mL of ice‐cold PBS was injected into the peritoneal cavity, massaged and cell suspension was aspirated. Cells were stained with trypan blue (1:1, Bio‐Rad) and counted using a TC‐20 automated cell counter (Bio‐Rad, USA). Cells were transferred to low glucose DMEM (Sigma–Aldrich, #D6046) supplemented with 10 % heat‐inactivated FBS and 1% penicillin–streptomycin and cultured in 48‐well plates at a plating density of 0.2 × 10^6^ cells/well and a total volume of 500 μL/well. Cells were incubated in the presence of *B. vulgatus* or *E. coli* LPS or with media only at +37°C, 5% CO_2_ for 20 h. LPS concentrations of 1, 0.1, 0.01 and 0.001 μg/mL were used. After 20 h, supernatants were collected and TNFα levels analysed. Culture media levels of TNFα were determined with ELISA MAX Deluxe Set Mouse TNF‐α (BioLegend, USA, #430904) according to the manufacturer’s protocol.

Female NOD mice ages of 5–8 weeks were injected intraperitoneally with 30 μg of *B. vulgatus* or *E. coli* LPS (priming), or PBS. Injection with PBS only served as a control. After 72 h, mice were killed, peritoneal cells harvested, and challenged in vitro as described above. The challenge was conducted with commercial *E. coli* LPS (*n* = 6 + 6 + 7) or zymosan A from *Saccharomyces cerevisiae* (Sigma–Aldrich, #58856‐93‐2) (*n* = 5 + 5 + 6) or with media only. Concentrations of commercial *E. coli* LPS: 0.1, 0.01 and 0.001 μg/mL. Concentrations of zymosan: 5 and 2.5 μg/mL. After 20 h, supernatants were collected and TNFα levels were determined as above.

### 4.5. DC Responses

A single 30 μg dose of either *E. coli* or *B. vulgatus* LPS or PBS was administered i.p. to female NOD mice of 5–6 weeks of age. 24 h (*n* = 5 per group) after dosing, mice were killed, PaLNs were collected and placed in RPMI‐1640 medium (Sigma–Aldrich, #R8758). RPMI + 10% FBS was added, and the tissues were passed through a metal strainer. Cells were filtered through a nylon membrane (77 μm), transferred into stain buffer (PBS, 2% FBS, 0.01% NaN_3_) and cell frequencies were determined as above. 2 × 10^6^ cells/animal were stained for 15 min at 4°C (panel as Table S1) and transferred into running buffer (PBS + 0.01% NaN_3_). The DC responses were analysed with NovoCyte (Agilent) flow cytometer and NovoExpress software (Agilent, USA), using the gating strategy in Figure S2.

About 4‐week‐old male NOD mice (*n* = 5 per group) were injected i.p. with two 30 μg doses of *B. vulgatus* or *E. coli* LPS or PBS on Days 0 and 3 (priming) and subsequently with a 30 μg dose of *E. coli* LPS (challenge) on Day 8. Mice were killed on Day 9 and PaLN DC populations were analysed with flow cytometry as above.

### 4.6. T Cell Responses

Two (priming and challenge) 5 μg doses of *B. vulgatus* or *E. coli* LPS or PBS were administered i.p. on Days 0 and 7 to female NOD mice (*n* = 6 per group) 4 weeks of age. Mice were killed on Day 15 and PaLNs were collected and cells isolated and treated for flow cytometry as described above. 2 × 10^6^ cells/animal were stained using markers in Table S2. T cells recognising the islet antigen IGRP were detected by using IGRP_206–214_ (LYLVCGERG) peptides in complex with H_2_K(d) MHC tetramers, provided by Emory University NIH Tetramer Core Facility (RRID:SCR_026557). Tetramer dilution (1:500) was added per 2 × 10^6^ cells. Cells were incubated for 30 min at 4°C and washed. Cell populations were analysed with flow cytometry as above.

### 4.7. CXCL10 Expression in Pancreatic Islets

About 4‐week‐old female mice (*n* = 7 per group) were treated i.p. with 30 μg of LPS or PBS. Pancreases were harvested after 72 h and snap frozen in liquid nitrogen. Frozen pancreases were embedded in Tissue‐Tek O.C.T. Compound (Sakura Finetek, #6200). Tissue blocks were cut into 6 μm sections with cryomicrotome at −25°C and placed onto Superfrost Plus Adhesion Microscope Slides (epredia). Sections were thawed and fixed in acetone for 5 min at −20°C. Blocking was done with 5% BSA (Sigma–Aldrich, #A9418‐50G) in PBS. The sections were stained with primary antibody anti m‐CXCL10/IP‐10 goat IgG (R&D Systems Inc., #AF‐466‐NA) for 50 min and with secondary antibody Alexa Fluor 555 donkey anti‐goat IgG (H + L; Invitrogen, #A‐21432) for 30 min. Sections were mounted with ProLong Diamond Antifade Mountant (Invitrogen, #P36965) and coverglass. Slides were cured overnight and imaged with a Nikon Eclipse Ti2‐E fluorescent microscope (Japan) using NIS Elements imaging software (version 5.11.03) and a Hamamatsu Orca C13440 Flash4.0 ERG (b/w) ccd camera (Japan). Data analysis was performed with ImageJ software.

About 4‐week‐old female mice (*n* = 7 per group) received two (priming and challenge) 5 μg i.p. injections of *B. vulgatus* or *E. coli* LPS or PBS on Days 0 and 7. Mice were killed on Day 15 and CXCL10 expression in pancreatic islets was studied as above.

### 4.8. Islet Inflammation

About 4‐week‐old female mice (*n* = 6 per group) received two 5 μg i.p. injections of *B. vulgatus* or *E. coli* LPS or PBS on Days 0 and 7. Mice were killed on Day 15 and mononuclear cell infiltration in pancreatic islets was quantified. Insulitis was scored using cryopreserved sections (6 μm) of pancreas stained with H&E. At least 60 individual islets per pancreas were scored as follows: 0, no insulitis; 1, peri‐insulitis with minimal infiltration; 2, insulitis with <50% infiltration; 3, invasive insulitis with >50% infiltration.

### 4.9. Statistical Analysis

All data were analysed with GraphPad Prism, excluding quantification of LPS concentration determined with Microsoft Excel. Outliers were detected with Tukey’s 1.5 IQR method and excluded from data. Normally distributed data were analysed with unpaired two‐tailed Student’s *t*‐test, as well as with one‐way ANOVA and Tukey’s multiple comparison, data not following Gaussian distribution with Mann–Whitney *U* test, as well as Kruskal–Wallis test and Dunn’s multiple comparison. LPS and TNFα concentrations were determined by removing baseline absorbance and fitting values to a linear regression curve. Normalisation between experiments was conducted on TNFα by adjusting TNFα release from control mice at the same level. CXCL10 expression in islets was determined with ImageJ. Results are presented as arbitrary units (the mean grey value of a pixel inside the ROI, a.k.a. the islet), calculated based on the sampled islets after subtracting background fluorescence. Normalisation between experiments was conducted by staining and imaging the same sample in all sets.

## Author Contributions

All authors contributed to the conception of study, acquisition of data, drafting or reviewing the manuscript and approved the final version to be submitted. Satu M. Silojärvi wrote the manuscript and researched data. Linda A. A. Leino researched data. Sakari A. Pöysti reviewed the manuscript and Arno L. M. Hänninen contributed to discussion and reviewed/edited the manuscript.

## Funding

The study was supported by Novo Nordisk Fonden (Grant NNF18OC0033880), the InFLAMES Flagship Programme of the Academy of Finland (Grant 337530), the state research funding for University Level Health Research in Turku University Hospital, Instrumentariumin Tiedesäätiö (Grant 200056), Emil Aaltosen Säätiö (Grant 210181), Diabetestutkimussäätiö (Grant 240022), Turun Yliopistosäätiö (Grant 081775). Open access publishing facilitated by Turun yliopisto, as part of the Wiley ‐ FinELib agreement.

## Conflicts of Interest

The authors declare no conflicts of interest.

## Supporting Information

Additional supporting information can be found online in the Supporting Information section.

## Supporting information


**Supporting Information** Table S1: Antibodies used in dendritic cell flow cytometry. Table S2: Antibodies used in T cell flow cytometry. Figure S1: Study timeline depicting exposures, measured responses and used methods. Figure S2: Dendritic cell gating strategy.

## Data Availability

The data that support the findings of this study are available from the corresponding author upon reasonable request.
