# Supplementary material for: Endotoxin Tolerance Impinges on T Cell Activation and Chemoattraction in Autoimmune Diabetes
Source: J Immunol Res. 2026 Apr 24;2026:7395567. doi: 10.1155/jimr/7395567 (PMC13107548; doi:10.1155/jimr/7395567)
Supplement: Supplementary file 1 — Supporting Information Table S1: Antibodies used in dendritic cell flow cytometry. Table S2: Antibodies used in T cell flow cytometry. Figure S1: Study timeline depicting exposures, measured responses and used methods. Figure S2: Dendritic cell gating strategy. [file JIMR-2026-7395567-s001.pdf]

SUPPLEMENTARY TABLES

Supplementary table 1. Antibodies used in dendritic cell flow cytometry.

Name	Clone	Manufacturer	#CATNo.	Dilution
Brilliant Violet 785™ anti-mouse CD45	30-F11	BioLegend®	103149	1:300
PE/Dazzle™ 594 anti-mouse CD3	17A2	BioLegend®	100246	1:300
PE/Dazzle™ 594 anti-mouse CD19	6D5	BioLegend®	115554	1:300
PE anti-rat RT1B	OX-6	BioLegend®	205308	1:300
Brilliant Violet 605™ anti-mouse CD11c	N418	BioLegend®	117334	1:300
Brilliant Violet 650™ anti-mouse/rat XCR1	ZET	BioLegend®	148220	1:300
APC/Fire™ 750 anti-mouse CD86	GL-1	BioLegend®	105046	1:300
APC anti-mouse CD80	16-10A1	BioLegend®	104714	1:300
FITC anti-mouse CD40	HM40-3	BioLegend®	102906	1:300
Zombie NIR™ Fixable Viability Kit (PE/Dazzle)		BioLegend®	423105	1:1000

Antibodies were diluted in FACS I buffer with 5% rat serum.

Supplementary table 2. Antibodies used in T cell flow cytometry.

Name	Clone	Manufacturer	#CATNo.	Dilution
Allophycocyanin (APC) 42692-IGRP		NIH Tetramer Reagent Core		1:500
Phycoerythrin (PE) 42690-control tetramer		NIH Tetramer Reagent Core		1:500
Brilliant Violet 785™ anti-mouse CD45	30-F11	BioLegend®	103149	1:300
PE/Cyanine5 anti-mouse CD4	GK1.5	BioLegend®	100410	1:300
APC/Cyanine7 anti-mouse CD8a	53-6.7	BioLegend®	100714	1:300
Brilliant Violet 510™ anti-mouse/human CD44	IM7	BioLegend®	103043	1:300
FITC anti-mouse CD69	H1.2F3	BioLegend®	104505	1:300
Brilliant Violet 421™ anti-mouse CD183 (CXCR3)	CXCR3-173	BioLegend®	126521	1:300

Antibodies and tetramers were diluted in FACS I buffer with 5% rat serum.

SUPPLEMENTARY FIGURES

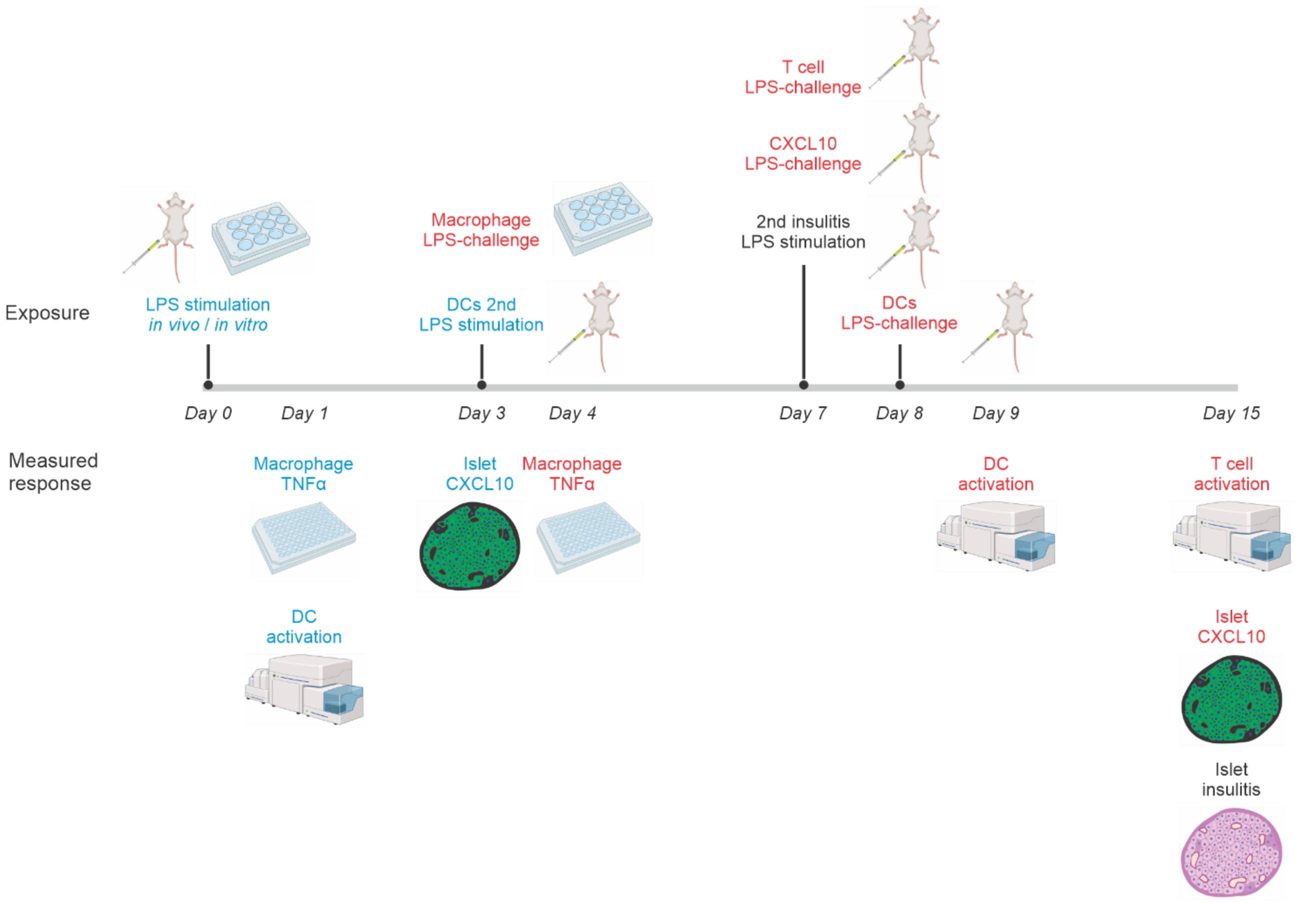

Supplementary figure 1. Study timeline depicting exposures, measured responses and used methods. Blue text indicates stimulatory doses of LPS and responses measured after stimulation, red text LPS-challenge and responses after challenge.

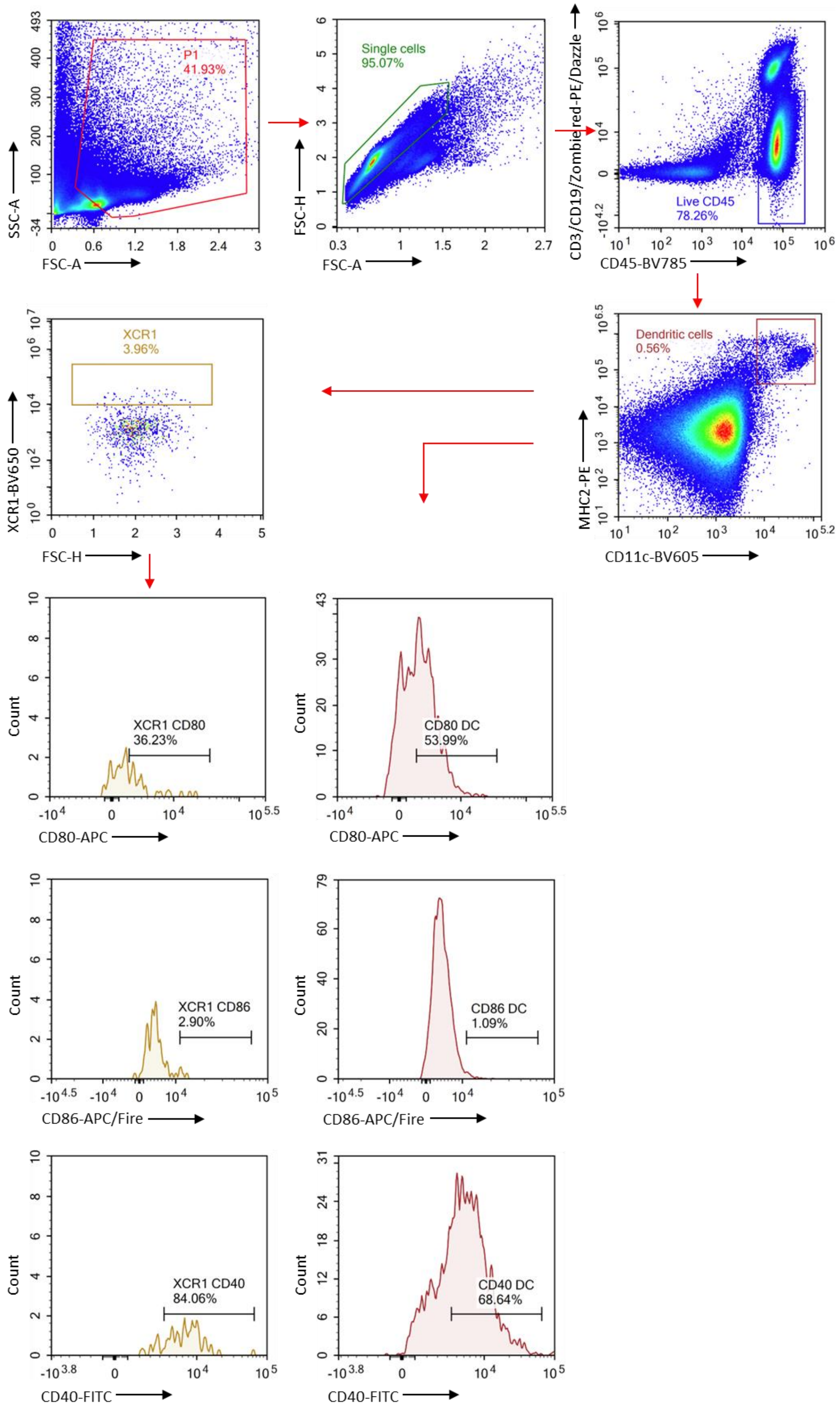

Supplementary figure 2. Dendritic cell gating strategy.
